# The association of spinal morning stiffness with lumbar disc degeneration and C-reactive protein: The back complaints in older adults (BACE) study

**DOI:** 10.1016/j.ocarto.2024.100535

**Published:** 2024-10-18

**Authors:** Daniel Feller, Roxanne van den Berg, Wendy T.M. Enthoven, Edwin H.G. Oei, Sita M. Bierma-Zeinstra, Bart W. Koes, Alessandro Chiarotto

**Affiliations:** a Department of General Practice, Erasmus MC, University Medical Center, Rotterdam, the Netherlands; b ConsultAssistent, Rotterdam, the Netherlands; c Department of Radiology & Nuclear Medicine, Erasmus MC, University Medical Center, Rotterdam, the Netherlands; d Department of Orthopedics and Sports Medicine, Erasmus MC, University Medical Center, Rotterdam, the Netherlands; e Research Unit of General Practice, Department of Public Health and the Center for Muscle and Joint Health, University of Southern Denmark, Odense, Denmark

**Keywords:** Spinal stiffness, Diagnostic criteria, Diagnosis, Back pain, Spinal osteoarthritis, Facet joint osteoarthritis

## Abstract

**Objective:**

To determine the association between patient-reported spinal morning stiffness and lumbar disc degeneration (LDD) and systemic inflammation, as measured by C-reactive protein (CRP), in older patients with non-specific back pain. The ultimate objective is to help shape a future definition of spinal osteoarthritis (OA).

**Design:**

Baseline data from the Dutch “Back Complaints in the Older Adults” (BACE) study was used. The relationship between the severity and duration of patient-reported spinal morning stiffness, LDD (i.e., multilevel disc space narrowing and multilevel osteophytes), and CRP was assessed. Regression models adjusted for confounding variables were performed.

**Results:**

Six hundred and seventy-five patients were included. The mean age was 66.52 years (SD 7.69), with a mean CRP of 3.20 ​mg/L (SD 7.61). The severity of spinal morning stiffness was associated with multilevel disc space narrowing: OR 2.89 (95 ​% CI: 1.24 to 6.74) for ‘mild’, OR 2.97 (95 ​% CI: 1.18 to 7.44) for ‘moderate’, OR 3.23 (95 ​% CI: 1.17 to 8.90) for ‘severe’, and OR 5.62 (95 ​% CI: 1.70 to 18.60) for ‘extreme’ morning stiffness severity. However, spinal morning stiffness severity was not associated with multilevel osteophytes, and both multilevel features of LDD showed no associations with the duration of spinal morning stiffness. No associations were found between spinal morning stiffness severity or duration, and CRP levels.

**Conclusions:**

Our results suggest that the severity of patient-reported spinal morning stiffness might be considered in future definitions of symptomatic spinal OA and that spinal morning stiffness is probably a symptom of a degenerative process in the spine rather than a symptom of systemic inflammation in patients with back pain.

## 1 Introduction

The mean lifetime prevalence of back pain is about 40 ​%, and, in primary care, it is the most common musculoskeletal complaint [1,2]. While mild back pain becomes less prevalent with age, severe back pain and co-morbidity tend to increase in older adults [[3], [4], [5]]. The prevalence of disc degeneration, spinal stenosis, osteoporosis, and facet joint osteoarthritis (OA) is known to increase with age, and these conditions can influence the course of back pain in older adults [[6], [7], [8]]. Notably, the increased prevalence of back pain in older adults may be attributed to a higher prevalence of lumbar disc degeneration (LDD) in this population, especially to its feature of multilevel disc space narrowing [6]. Features of LDD, like osteophytes and disc space narrowing, are considered radiographic signs of spinal OA [9,10]. Nevertheless, we currently lack definitions of structural or symptomatic spinal OA, and recently there have been calls to define this condition to improve clinical outcomes in patients with back pain [11,12].

Defining spinal OA and creating diagnostic criteria for this condition requires determining the association between clinical signs and symptoms of back pain, and radiographic features of OA. A recent Delphi study reached consensus that *“self-reported morning stiffness in the spine should be considered in a symptomatic definition of spinal OA, for both research purposes and clinical practice”*. [11] Also, a systematic review found high-quality evidence of an association between patient-reported spinal morning stiffness and disc space narrowing on radiographs in patients with back pain, and moderate-quality evidence of an association between spinal morning stiffness and osteophytes on radiographs [13]. However, spinal morning stiffness could also be caused by a systemic inflammatory process. In fact, morning stiffness is part of the early referral recommendation for newly diagnosed rheumatoid arthritis [14]. Elevated levels of (pro-)inflammatory biomarkers, such as C-reactive protein (CRP), have also been reported in back pain patients [15], suggesting a possible systemic inflammatory cause.

Considering that two previous studies found associations between spinal morning stiffness and radiographic features of spinal OA [13], it would be possible to use this symptom to identify patients with back-related symptoms caused by spinal OA. However, spinal morning stiffness in older patients with back pain might also be a symptom of arthritis-related systemic inflammation. Therefore, this study aimed at: 1) re-assessing the association between patient-reported spinal morning stiffness (i.e., severity and duration) and radiographic features of LDD; 2) evaluating whether there is an association between spinal morning stiffness (i.e., severity and duration) and CRP (a biomarker of systemic inflammation).

## 2 Method

This study is reported according to the “STrengthening the Reporting of OBservational studies in Epidemiology” (STROBE) statement [16].

### 2.1 Study design

For this cross-sectional study, baseline data from the Back Complaints in the Older Adults (BACE) study was used. BACE was a prospective observational cohort study performed in the Netherlands [17]. The BACE study was approved by the Erasmus MC medical ethical committee (registration no.: NL24829.078.08) on March 23, 2009. Between March 2009 and September 2011, 675 patients aged >55 who visited their general practitioner with a new episode of back pain (i.e., they did not consult the general practitioner in the preceding six months because of back pain) were included. Back pain could be located from the top of the shoulder blades to the first sacral vertebra. Exclusion criteria were: patients unable to fill in questionnaires (due to language problems or cognitive disorders) and patients unable to undergo a physical examination (e.g., patients in a wheelchair).

### 2.2 Measurements

Baseline measurements included questionnaires, blood samples, physical examination, and lumbar spine radiographs. At baseline, the following items were assessed with questionnaires [4].

- General characteristics: age, gender, body mass index (BMI), educational level, and marital status [18,19].
- Characteristics of the back complaint: duration of back pain in days; severity of back pain in the last week measured on an 11-point numeric rating scale, with 0 representing ‘no pain’ and 10 representing the ‘worst imaginable pain’ [20,21]; physical functioning was measured with the Roland Disability Questionnaire (RMDQ scale, range 0–24) [22].
- Medical consumption: use of pain medication for back pain and treatment from a physical therapist.
- Psychological factors: depressive symptoms measured with the Center for Epidemiologic Studies Depression Scale (CES-D: score range 0–60) [23], kinesiophobia measured with the Fear-Avoidance Beliefs Questionnaire (FABQ: score range 0–28) [24], and pain catastrophizing measured with the Pain Catastrophizing scale (PCS: score range 0–52) [25].

### 2.3 Spinal morning stiffness

When evaluating the severity of patient-reported spinal morning stiffness, patients were asked “How severe is the stiffness in your back after waking up in the morning?”. Patients rated their stiffness on a 5-point scale, with 0 indicating ‘no morning stiffness', 1 indicating ‘mild spinal morning stiffness’, 2 indicating ‘moderate spinal morning stiffness’, 3 indicating ‘severe spinal morning stiffness’, and 5 indicating ‘extreme spinal morning stiffness'. To measure the duration of spinal morning stiffness, patients were asked to report the duration of their stiffness after waking up in the morning from two options: ‘spinal morning stiffness lasting less than 30 ​min’, or ‘spinal morning stiffness lasting more than 30 ​min’.

### 2.4 Systemic inflammation

Systemic inflammation was quantified with the concentration of CRP in the blood (measured in mg/L) [26]. At baseline, venous blood was drawn by a trained nurse; then, blood samples were centrifuged and stored at −20 C^o^. An independent laboratory assistant measured the concentration of CRP. The serum level of CRP was determined using a nephelometric method on the Image 800. In the case of a group of CRP below 1 ​mg/L, we assumed this variable to be equal to 0.

### 2.5 Lumbar radiographs

A lateral and antero-posterior weight-bearing lumbar spine radiograph was taken in the hospital nearest to the patient. The radiographs were scored for two LDD features: i) osteophytes and ii) disc space narrowing. The presence of these features was evaluated per disc level using the four grades of the Lane Atlas [12,27], i.e. grade 0 ​= ​none, grade 1 ​= ​mild, grade 2 ​= ​moderate and grade 3 ​= ​severe. Due to the low prevalence of grade 0 osteophytes in this study population (3 ​%), grade 0 and grade 1 osteophytes were combined. In the present study, the following radiographic definitions of ‘osteophytes’ and ‘disc space narrowing’ were used [6].

- Multilevel ​≥ ​grade 2 osteophytes, defined as ​≥ ​grade 2 osteophytes at two or more levels for L1-2 to L5-S1.
- Multilevel ​≥ ​grade 1 disc space narrowing, defined as ​≥ ​grade 1 disc space narrowing at two or more levels from L1-2 to L5-S1.

All lumbar radiographs were independently scored for the features of LDD by two observers. These two observers were trained by an experienced musculoskeletal radiologist and blinded to the clinical characteristics of the patients. The interrater agreement was substantial (Kappa 0.71) [28].

### 2.6 Statistical analysis

Descriptive statistics were used to present the characteristics of the included patients. Baseline characteristics were compared using Wilcoxon and Pearson tests for continuous and categorical variables, respectively, between those with or without LDD.

The association of multilevel ​≥ ​grade 1 disc space narrowing with the severity and duration of morning stiffness was assessed using a multivariable logistic regression model adjusted for age, gender, BMI, back pain severity, duration of back pain (in days), history of previous back surgery, and CRP level. We also applied the same model to determine the association of multilevel ​≥ ​grade 2 osteophytes with the severity and duration of morning stiffness. Furthermore, we evaluated the relationship between morning spinal stiffness (severity and duration) and systemic inflammation (indicated by CRP level) using a multivariable ordinal logistic model adjusted for age, gender, BMI, back pain severity, duration of back pain (in days), and history of previous back surgery [29]. In all analyses, we assumed a smooth relationship of the continuous covariates using restricted cubic regression splines with three knots placed at the 10th, 50th, and 90th percentile. The primary analysis was conducted on imputed datasets. As a sensitivity analysis, we also performed all multivariable models using a complete-cases approach. Statistical analyses were performed using R with the rms, Hmisc, MASS, mice, lubridate, VIM, and corrplot packages [[29], [30], [31]]. Supplementary 1 reports additional information on the statistical analysis conducted.

## 3 Results

### 3.1 Patients’ characteristics

Table 1 presents patients' characteristics. The mean age of patients was 66.52 years (SD 7.69), and 59 ​% were females. The mean RMDQ was 9.78 (SD 5.82), with mean pain intensity in the previous week of 5.16 (SD 2.68). Among the included individuals, 27 ​% had multilevel ​≥ ​grade 1 disc space narrowing, and 41 ​% had multilevel ​≥ ​grade 2 osteophytes. Most patients (i.e., 56 ​%) reported their spinal morning stiffness as “moderate” or “severe”. Among the patients with spinal morning stiffness, 24 ​% reported experiencing it for more than 30 ​min. Patients with LDD differed significantly from those without multilevel degeneration. For instance, the duration of morning stiffness was statistically different (p ​< ​0.01) between individuals with or without LDD.

**Table 1:** Characteristics at baseline of older adults with back pain (BACE cohort, missing values excluded).

	Overall	Multilevel ​≥ ​grade 1 disc space narrowing			Multilevel ​≥ ​grade 2 osteophytes		
		Not present N ​= ​460	Present N ​= ​173	P-value	Not present N ​= ​374	Present N ​= ​259	P-value
**Age**							
	66.52 (7.69)	65.44 (7.18)	69.03 (7.94)	**< 0.01**	65.39 (7.30)	67.92 (7.69)	**< 0.01**
**Gender**							
Females	396 (0.59)	25 (0.55)	118 (0.68)	**< 0.01**	243 (0.65)	126 (0.49)	**< 0.01**
Males	275 (0.41)	207 (0.45)	55 (0.32)		130 (0.35)	132 (0.51)	
**BMI**							
	27.5 (4.68)	27.59 (4.80)	27.13 (4.35)	0.28	27.12 (4.65)	27.95 (4.70)	**0.02**
**Educational level**							
Low	464 0.69)	309 (0.65)	125 (0.71)	**0.03**	261 (0.71)	173 (0.68)	**< 0.01**
Middle	90 (0.14)	63 (0.14)	23 (0.14)		52 (0.13)	34 (0.13)	
High	114 (0.17)	85 (0.19)	25 (0.15)		60 (0.16)	50 (0.19)	
**Marital status**							
Single	47 (0.07)	28 (0.06)	17 (0.10)	**< 0.01**	23 (0.06)	22 (0.09)	0.43
Married	479 (0.73)	348 (0.76)	107 (0.62)		280 (0.76)	175 (0.68)	
Divorced	45 (0.06)	27 (0.06)	15 (0.09)		23 (0.06)	19 (0.07)	
Widowed	73 (0.10)	42 (0.09)	23 (0.13)		35 (0.09)	30 (0.12)	
Living together	24 (0.04)	12 (0.03)	11 (0.06)		12 (0.03)	11 (0.04)	
**RMDQ**							
	9.78 (5.82)	9.52 (5.89)	10.35 (5.58)	0.13	9.42 (5.84)	10.22 (5.75)	0.10
**Mean pain last week (NRS 0**–**10)**							
	5.16 (2.68)	5.00 (2.69)	5.51 (2.65)	**0.03**	4.94 (2.73)	5.43 (2.61)	**0.03**
**Number of days with pain**							
	248.3 (974.4)	212.1 (909.4)	365.1 (1221.9)	**< 0.01**	193.4 (716.4)	341.3 (1319.9)	0.17
**Frequency of pain**							
Less than once a week	80 (0.12)	63 (0.14)	14 (0.08)	0.30	49 (0.13)	28 (0.11)	0.04
At least once a week	21 (0.03)	16 (0.04)	5 (0.03)		15 (0.04)	6 (0.02)	
Every day, at least a few minutes	178 (0.27)	121 (0.27)	48 (0.29)		108 (0.29)	61 (0.24)	
Every day for most of the day	310 (0.48)	208 (0.46)	80 (0.48)		169 (0.47)	119 (0.49)	
Every minute of the day	65 (0.10)	41 (0.09)	21 (0.12)		27 (0.07)	35 (0.14)	
**Back pain in the past**							
Yes	579 (0.87)	386 (0.84)	157 (0.92)	**0.02**	316 (0.85)	227 (0.88)	0.35
No	87 (0.13)	71 (0.16)	14 (0.08)		54 (0.15)	31 (0.12)	
**Previous back surgery**							
Yes	56 (0.08)	32 (0.07)	23 (0.13)	**0.01**	31 (0.08)	24 (0.09)	0.66
No	608 (0.92)	422 (0.93)	149 (0.87)		339 (0.92)	232 (0.91)	
**Morning stiffness duration**							
None	163 (0.25)	123 (0.27)	32 (0.19)	**< 0.01**	95 (0.26)	60 (0.23)	**< 0.01**
Less than 30 ​min	338 (0.51)	242 (0.53)	82 (0.48)		208 (0.56)	116 (0.46)	
More than 30 ​min	160 (0.24)	90 (0.20)	55 (0.33)		65 (0.18)	80 (0.31)	
**Morning stiffness severity**							
None	92 (0.14)	74 (0.16)	13 (0.08)	**< 0.01**	56 (0.15)	31 (0.12)	0.43
Mild	155 (0.23)	111 (0.24)	39 (0.23)		95 (0.25)	55 (0.21)	
Moderate	229 (0.34)	156 (0.35)	58 (0.33)		119 (0.32)	95 (0.38)	
Severe	147 (0.22)	92 (0.20)	45 (0.26)		77 (0.21)	60 (0.23)	
Extreme	45 (0.07)	24 (0.05)	18 (0.10)		26 (0.07)	16 (0.06)	
**Drug treatment**							
Yes	483 (0.73)	332 (0.73)	123 (0.71)	0.67	276 (0.74)	179 (0.70)	0.21
No	183 (0.27)	124 (0.27)	50 (0.39)		96 (0.26)	78 (0.30)	
**Physiotherapy treatment**							
Yes	299 (0.45)	204 (0.45)	76 (0.45)	0.92	162 (0.44)	118 (0.46)	0.65
No	359 (0.55)	248 (0.55)	94 (0.55)		204 (0.56)	138 (0.54)	
**FABQ**							
	33.64 (20.35)	32.8 (19.6)	37.2 (21.7)	0.07	32.6 (19.6)	36.1 (21.1)	0.10
**PCS**							
	14.1 (10.57)	13.5 (10.1)	15.0 (11.2)	0.19	13.3 (10.4)	14.8 (10.4)	**0.04**
**CES-D**							
	13.89 (5.93)	14.04 (5.54)	13.61 (6.82)	0.39	14.34 (5.49)	13.33 (6.44)	**< 0.01**
**CRP**							
	3.20 (7.61)	3.02 (5.85)	3.00 (6.91)	0.99	3.03 (6.89)	3.00 (4.92)	0.33
**Multilevel disc space narrowing**							
Yes	173 (0.27)	–	–	–	54 (0.14)	119 (0.46)	**< 0.01**
No	460 (0.73)				320 (0.86)	140 (0.54)	
**Multilevel osteophytes**							
Yes	259 (0.41)	140 (0.30)	119 (0.69)	**< 0.01**	–	–	–
No	374 (0.59)	320 (0.70)	54 (0.31)				

Categorical variables are expressed as absolute frequency (relative frequency). Continuous variables are expressed as mean (standard deviation).

RMDQ: Roland Morris Disability Questionnaire (range 0–24), NRS: Numeric Rating Scale (range 0–10), FABQ: Fear-Avoidance Beliefs Questionnaire (range 0–96), PCS: Pain Catastrophizing scale (range 0–52), CES-D: Center for Epidemiologic Studies Depression Scale (range 0–60), CRP: C-reactive Protein.

Of the 675 patients included at baseline, the CRP level was the most commonly missing variable (8 ​%). In 43 cases (6.3 ​%), it was not possible to determine the presence of both multilevel ​≥ ​grade 1 disc space narrowing and multilevel ​≥ ​grade 2 osteophytes due to one or more missing radiograph evaluations. Patients with a missing value for LDD differed from those with a non-missing value in their mean CRP level (p–value: 0.01) (see Supplementary 2).

### 3.2 Association of multilevel ≥ grade 1 disc space narrowing with the severity and duration of spinal morning stiffness

In the univariable unadjusted analysis, spinal morning stiffness severity was statistically associated with multilevel ​≥ ​grade 1 disc space narrowing. Notably, increasing reported severity was associated with a higher risk of LDD (Table 2). The results of the multivariable adjusted regression showed that spinal stiffness severity was still associated with multilevel ​≥ ​grade 1 disc space narrowing: OR 2.89 (95 ​% CI: 1.24 to 6.74) for ‘mild’, OR 2.97 (95 ​% CI: 1.18 to 7.44) for ‘moderate’, OR 3.23 (95 ​% CI: 1.17 to 8.90) for ‘severe’, and OR 5.62 (95 ​% CI: 1.70 to 18.60) for ‘extreme’ morning stiffness severity (Table 2). However, the duration of spinal morning stiffness was not associated with multilevel ​≥ ​grade 1 disc space narrowing in the multivariable model (Table 2). The complete case analysis showed the same results, except for the ‘moderate’ and ‘severe’ category of spinal stiffness severity, which was not statistically associated with multilevel ​≥ ​grade 1 disc space narrowing (OR 2.46 with 95 ​% CI 0.90 to 6.90 and OR 2.20 with 95 ​% CI 0.73 to 6.79, respectively) (see Supplementary 3).

**Table 2:** Regression outputs for multilevel disc space narrowing and osteophytes.

	MULTILEVEL DISC SPACE NARROWING				MULTILEVEL OSTEOPHYTES			
	Univariable association (N ​= ​675)		Multivariable association (N ​= ​675) a		Univariable association (N ​= ​675)		Multivariable association (N ​= ​675) a	
	OR (95 ​% CI)	P-value	OR (95 ​% CI)	P-value	OR (95 ​% CI)	P-value	OR (95 ​% CI)	P-value
**Morning stiffness severity**								
Reference: None	**-**	**-**	**-**	**-**	**-**	**-**	**-**	**-**
Mild	**2.08 (1.03 to 4.22)**	**0.04**	**2.89 (1.24 to 6.74)**	**0.01**	1.06 (0.61–1.84)	0.83	1.11 (0.57–2.17)	0.76
Moderate	**2.21 (1.15 to 4.23)**	**0.02**	**2.97 (1.18 to 7.44)**	**0.02**	1.44 (0.84–2.47)	0.19	1.52 (0.70–3.31)	0.29
Severe	**2.89 (1.45 to 5.78)**	**< 0.01**	**3.23 (1.17 to 8.90)**	**0.02**	1.41 (0.81–2.46)	0.23	0.99 (0.43–2.28)	0.98
Extreme	**4.35 (1.87 to 10.13)**	**< 0.01**	**5.62 (1.70 to 18.60)**	**< 0.01**	1.08 (0.51–2.28)	0.83	0.58 (0.20–1.68)	0.32
**Morning stiffness duration**								
Reference: No stiffness	–	–	–	–	–	–	–	–
Less than 30 ​min	1.25 (0.78–1.99)	0.35	0.82 (0.43–1.58)	0.56	0.88 (0.59–1.30)	0.51	0.80 (0.45–1.42)	0.45
More than 30 ​min	**2.26 (1.31 to 3.91)**	**< 0.01**	1.09 (0.49–2.44)	0.82	**1.90 (1.19 to 3.02)**	**< 0.01**	1.75 (0.87–3.54)	0.12

a Adjusted for gender, body mass index (splines with 3 knots), age (splines with 3 knots), CRP (splines with 3 knots), mean pain last week (splines with 3 knots), duration of back pain (splines with 3 knots), and previous back surgery.

### 3.3 Association of multilevel ≥ grade 2 osteophytes with the severity and duration of spinal morning stiffness

The severity of spinal morning stiffness was found to have no association with multilevel osteophytes in both the univariable and multivariable analyses, as shown in Table 2. Morning stiffness duration of more than 30 ​min was associated with multilevel ​≥ ​grade 2 osteophytes in the univariable logistic regression (OR 1.90; 95 ​% CI: 1.19 to 3.02). This association was no longer statistically significant in the adjusted model (OR 1.75; 95 ​% CI: 0.87 to 3.54). The complete-case approach confirmed the primary analysis results. (see Supplementary 3).

### 3.4 Association of the presence of spinal morning stiffness with CRP levels

In both the adjusted and unadjusted analyses, the severity or the duration of morning stiffness was not associated with CRP levels (Table 3). The results were confirmed using a complete–case approach (see Supplementary 3).

**Table 3:** Regression output for the presence of morning stiffness.

	MORNING STIFFNESS SEVERITY				MORNING STIFFNESS DURATION			
	Univariable association (N ​= ​675)		Multivariable association (N ​= ​675) a		Univariable association (N ​= ​675)		Multivariable association (N ​= ​675) a	
	Coefficient (95 ​% CI)	P-value	Coefficient (95 ​% CI)	P-value	Coefficient (95 ​% CI)	P-value	Coefficient (95 ​% CI)	P-value
**CRP**^b^								
Spline	0.08 (−0.05 to 0.21)	0.21	0.06 (−0.08 to 0.19)	0.41	0.06 (−0.07 to 0.20)	0.35	0.02 (−0.12 to 0.17)	0.77
Spline’	−0.06 (−0.22 to 0.10)	0.47	−0.04 (−0.21 to 0.13)	0.66	−0.05 (−0.22 to 0.12)	0.57	−0.01 (−0.19 to 0.17)	0.92

CI: confidence interval; OR: odds ratio; CRP: C-reactive protein.

a Adjustement for mean pain last week (splines with 3 knots), gender, age (splines with 3 knots), body mass index (splines with 3 knots), duration of back pain (splines with 3 knots), and previous back surgery.

b Restricted cubic splines calculated using the “rcs” function of the “rms” package. Individual coefficients of the splines should not be interpreted. For a general interpretation of the output of the “rcs” function see Harrell's book “Regression modeling strategies”.

## 4 Discussion

This study is the first to evaluate the association between self-reported spinal morning stiffness and LDD in a cohort of older patients with back pain in primary care. Our findings suggest that the severity of spinal morning stiffness is associated with multilevel disc space narrowing but not with the presence of osteophytes at multiple levels (Table 2). Earlier, multilevel disc-space narrowing was shown to be the strongest LDD feature associated with low back pain in the general population of older adults [6]. At the same time, we found no association between the duration of spinal morning stiffness and LDD (Table 2). Furthermore, this is also the first study to investigate the relationship of spinal morning stiffness severity and duration with systemic inflammation, as measured by CRP, in older patients with non-specific back pain. We found no association between CRP levels and either the severity or duration of spinal morning stiffness (Table 3).

Our findings confirm the results of a previous study that established an association between the severity of spinal morning stiffness and multilevel disc space narrowing in the “Cohort Hip and Cohort Knee” (CHECK) data [32]. However, van den Berg et al. [33] also found an association between spinal morning stiffness severity and multilevel osteophytes, which our study did not replicate. Several factors might have influenced this varied outcome. For example, our analysis technique differed since we used LDD as the dependent variable, while van den Berg et al. [33] used spinal morning stiffness severity. Additionally, the inclusion criteria of the CHECK study were significantly different from the BACE cohort [32]: primary care patients were initially included for hip or knee OA and were aged 45–65 years old.

Our findings on spinal stiffness duration align with the study conducted by van den Berg et al. [33] However, they contradict the results of a previous study by Scheele et al. [34], which showed an association between LDD and the duration of spinal morning stiffness (multilevel narrowing: OR 1.4 with 95 ​% CI of 1.0–1.99; multilevel osteophytes: OR 2.6 with 95 ​% CI of 1.9–3.5). Even in this case, the different inclusion criteria used in the study by Scheele et al. [34] may have contributed significantly to the differing outcomes. Scheele et al. [34] used data from an open population cohort including above all participants without back pain. Additionally, the analysis was not adjusted for back pain intensity, duration of back pain, history of back surgery, and CRP.

Previous studies found an association between back pain presence/severity and inflammatory biomarkers such as CRP, with higher pain indicating higher levels of inflammation [15]. Nevertheless, this is the first study to assess the association between spinal morning stiffness and systemic inflammation, as measured by CRP, in patients with back pain. In the current study, spinal morning stiffness severity and duration were not associated with CRP levels, which most likely implies no systemic inflammatory component causing the spinal morning stiffness.

### 4.1 Implication for future research

It is important to conduct further studies on patients with back pain to confirm our research findings. Specifically, given the inconsistent evidence, we need additional studies investigating the possible association between the duration of spinal morning stiffness and LDD, and the relationship between the severity of spinal morning stiffness and the presence of osteophytes at multiple levels. Moreover, there is evidence of a positive relationship between inflammatory biomarkers, such as IL-6 and TNF-α, and back pain [15]. Kapoor et al. [35] review also suggests that IL-1β, TNF, and IL-6 are the primary inflammatory cytokines involved in OA. Therefore, future studies should examine the possible connection between other inflammatory biomarkers, spinal morning stiffness, and LDD. Finally, future studies should investigate the association of spinal morning stiffness with other structural spinal OA imaging features, such as facet joint abnormalities, Modic changes, and endplate lesions. To investigate these associations further, it would be preferable to use additional imaging techniques, such as magnetic resonance imaging and computed tomography scans.

### 4.2 Strengths and limitations

The present study has several strengths. First, validated questionnaires were used to measure clinical symptoms. Second, serum CRP was measured in a qualified laboratory. Third, the observers assessed the radiographs using standardized methods and were trained by an experienced musculoskeletal radiologist. Fourth, a biostatistician conducted all analyses using data from multiple imputations, with confirmed results from a sensitivity analysis using a complete case approach. Nevertheless, it is also worth noting that this study has some limitations. First, only the lateral and postero-anterior radiographs were used for the analysis, which could have led to an underestimation of the features of LDD. Second, we focused solely on disc space narrowing and osteophytes, evaluated through weight-bearing radiography, which is advantageous for these specific features. However, we did not assess other important characteristics of LDD, such as herniated discs, decreased water content, or annular tears, for which MRI would provide superior imaging. Future studies incorporating MRI could offer a more comprehensive evaluation of LDD and its association with morning stiffness*.* Third, the duration of spinal morning stiffness was measured as a categorical variable (more or less than 30 ​minutes). This approach limited the level of detail that could be obtained from this variable. If stiffness duration had been measured as a continuous variable, it would have been possible that the analysis would have revealed an association with LDD. Third, the LDD features were dichotomized, which made it impossible to distinguish between the severity of disc space narrowing and osteophytes. Finally, we measured CRP levels as a potential marker of systemic inflammation and found no association with this biomarker. However, we cannot rule out the possibility that there may be a connection between spinal morning stiffness and other biomarkers of systemic inflammation, such as Interleukin-6 and Tumor Necrosis Factor-Alpha.

### 4.3 Conclusions

Our research findings provide insights that might be considered in future definitions of symptomatic spinal OA. Particularly, this study confirmed an association between self-reported spinal morning stiffness severity and radiographic multilevel disc space narrowing in older patients with non-specific back pain. However, no association was found between spinal morning stiffness severity and multilevel osteophytes, spinal morning stiffness duration and both LDD features, and spinal morning stiffness and CRP. This last finding suggests that spinal morning stiffness is probably a symptom of a degenerative process in the spine rather than a symptom of systemic inflammation in patients with back pain.

## Author contributions

Daniel Feller: Conception and design, analysis and interpretation of the data, drafting of the article, critical revision of the article for important intellectual content, and final approval of the article.

Roxanne Van den Berg: Conception and design, critical revision of the article for important intellectual content, and final approval of the article.

Wendy Enthoven: Conception and design, critical revision of the article for important intellectual content, and final approval of the article.

Edwin Oei: Conception and design, critical revision of the article for important intellectual content, and final approval of the article.

Sita Bierma-Zeinstra: Conception and design, critical revision of the article for important intellectual content, and final approval of the article.

Bart Koes: Conception and design, critical revision of the article for important intellectual content, and final approval of the article.

Alessandro Chiarotto: Conception and design, critical revision of the article for important intellectual content, and final approval of the article.

Daniel Feller (d.feller@erasmusmc.nl) is responsible for the integrity of the work from its inception to the finished article.

## Declaration of Generative AI and AI-assisted technologies in the writing process

During the preparation of this work, the author(s) used Grammarly in order to paraphrase or simplify our own writing. After using this tool/service, the author(s) reviewed and edited the content as needed and take(s) full responsibility for the content of the publication.

## Role of the funding source

The BACE study was funded by the Department of General Practice, Erasmus MC, Rotterdam, Netherlands, and the Coolsingel Foundation, Rotterdam, The Netherlands. The BACE study was approved by the Erasmus MC medical ethical committee (registration no.: NL24829.078.08) on March 23, 2009.

The study's sponsor had no role in the study design, data collection, analysis, and interpretation, manuscript writing, or decision to submit the manuscript for publication.

## Declaration of competing interest

The authors declare no conflict of interest.
