# Supplementary material for: The association of spinal morning stiffness with lumbar disc degeneration and C-reactive protein: The back complaints in older adults (BACE) study
Source: Osteoarthr Cartil Open. 2024 Oct 18;6(4):100535. doi: 10.1016/j.ocarto.2024.100535 (PMC11566339; doi:10.1016/j.ocarto.2024.100535)
Supplement: Multimedia component 1 [file mmc1.docx]

**SUPPLEMENTARY MATERIAL 1 – STATISTICAL ANALYSIS**

Missing variables were handled by multiple imputations by chained equations, using logistic regressions for binary variables, Bayesian polytomous regressions for categorical variables with more than two categories, and predictive mean matching for continuous variables. ^1^ We utilized all the measurements mentioned in the methods section of the manuscript for the imputation model. Initially, we intended to exclude variables that had a high correlation (Spearman correlation coefficient > 0.9) or a high percentage of missing values (> 50%) from the imputation model. ^1^ However, after examining the variables, we found none exhibited these characteristics (Figure 1 and 2).


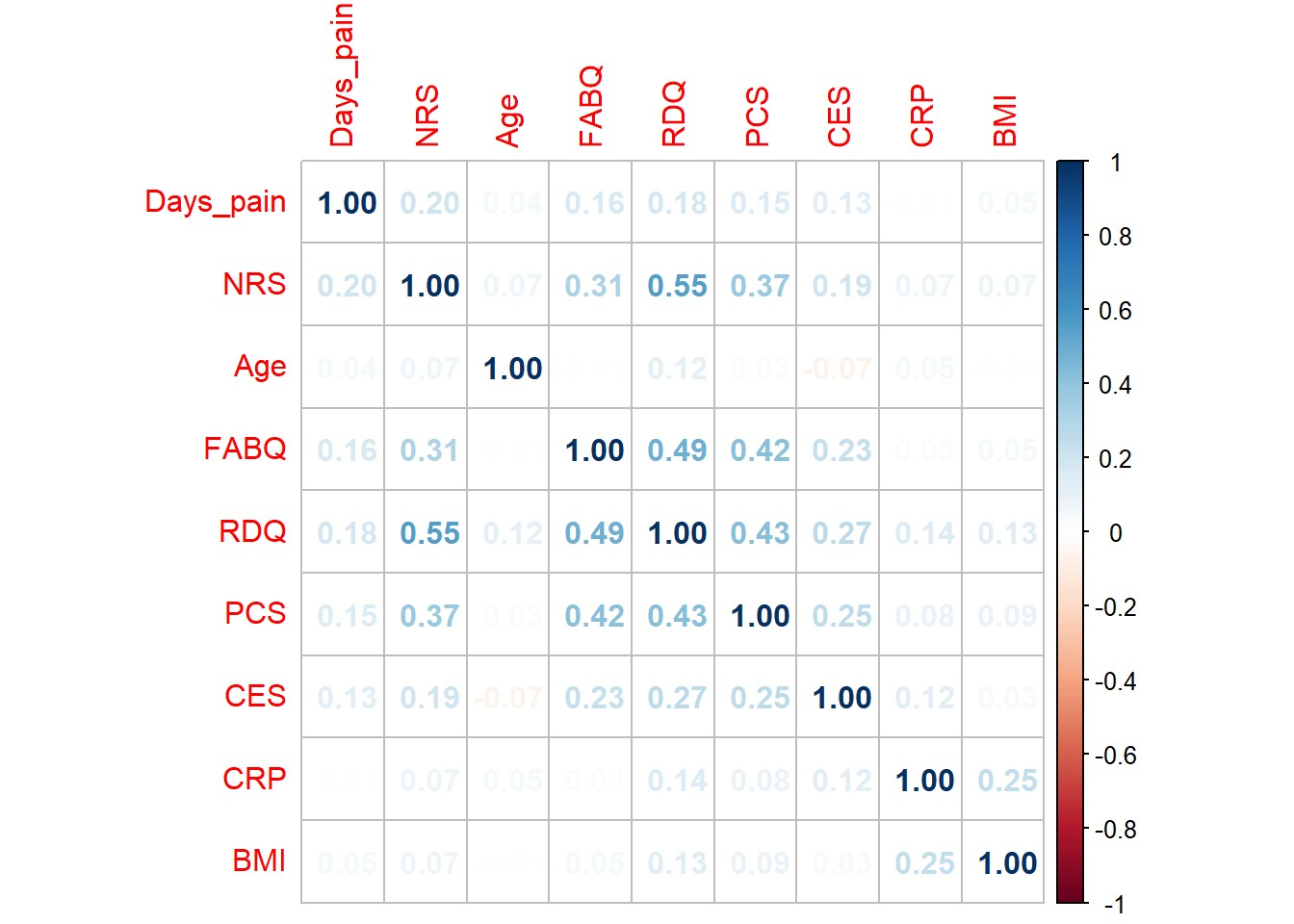


Figure 1 - Correlation plot


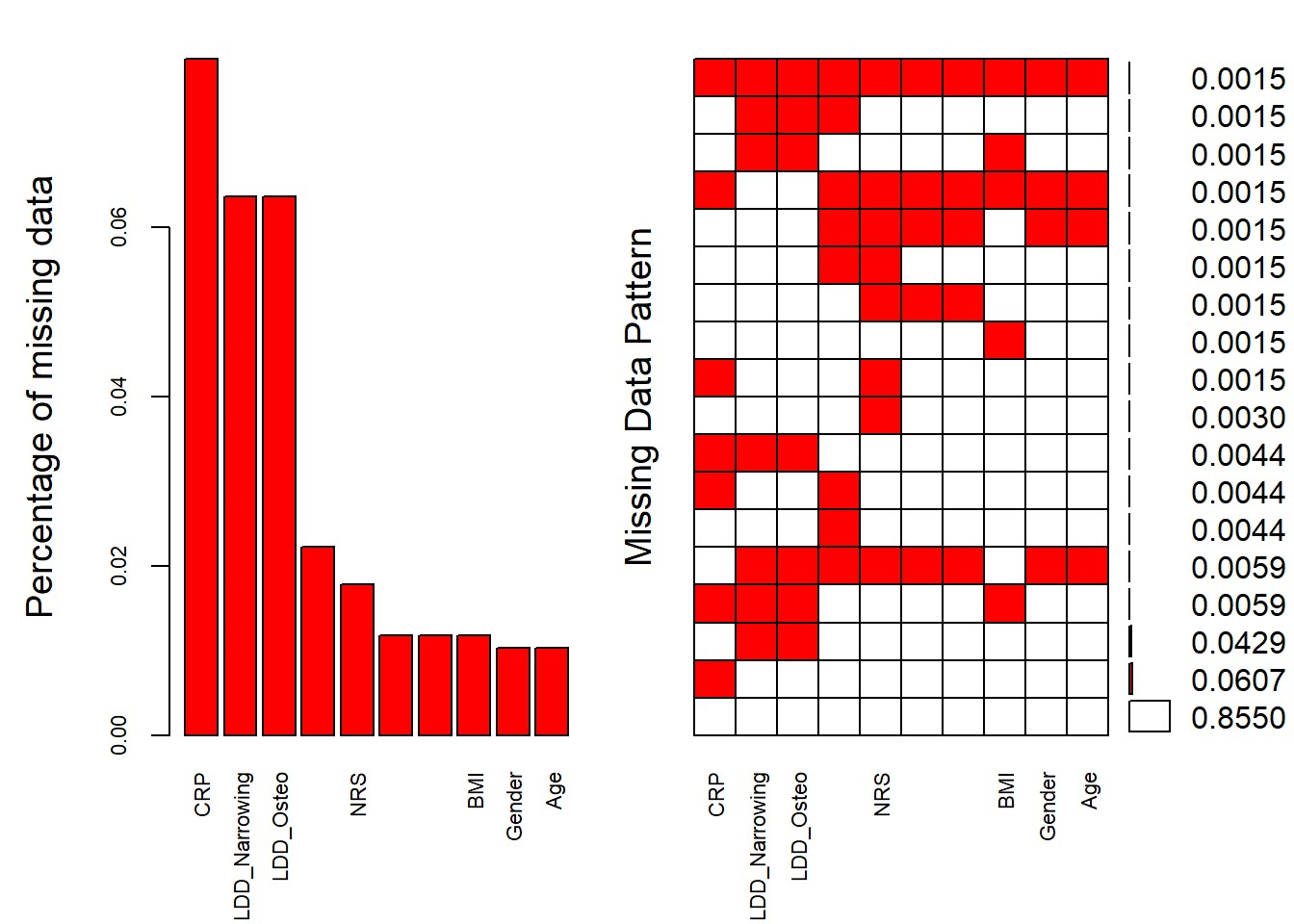


Figure 2 - Missing data's pattern

Based on the general rule that the number of imputed datasets should be equal to the percentage of the most missing variable (reactive C-protein: 8% missing), we imputed ten datasets using 20 iterations. ^1^ Convergence was assessed visually (Figure 3 to 10), and the imputed data was compared to the observed data to determine the plausibility of the imputed datasets (Figure 11). ^1^ The parameters of substantive interest were estimated in each imputed dataset separately and combined using Rubin’s rules. ^1^


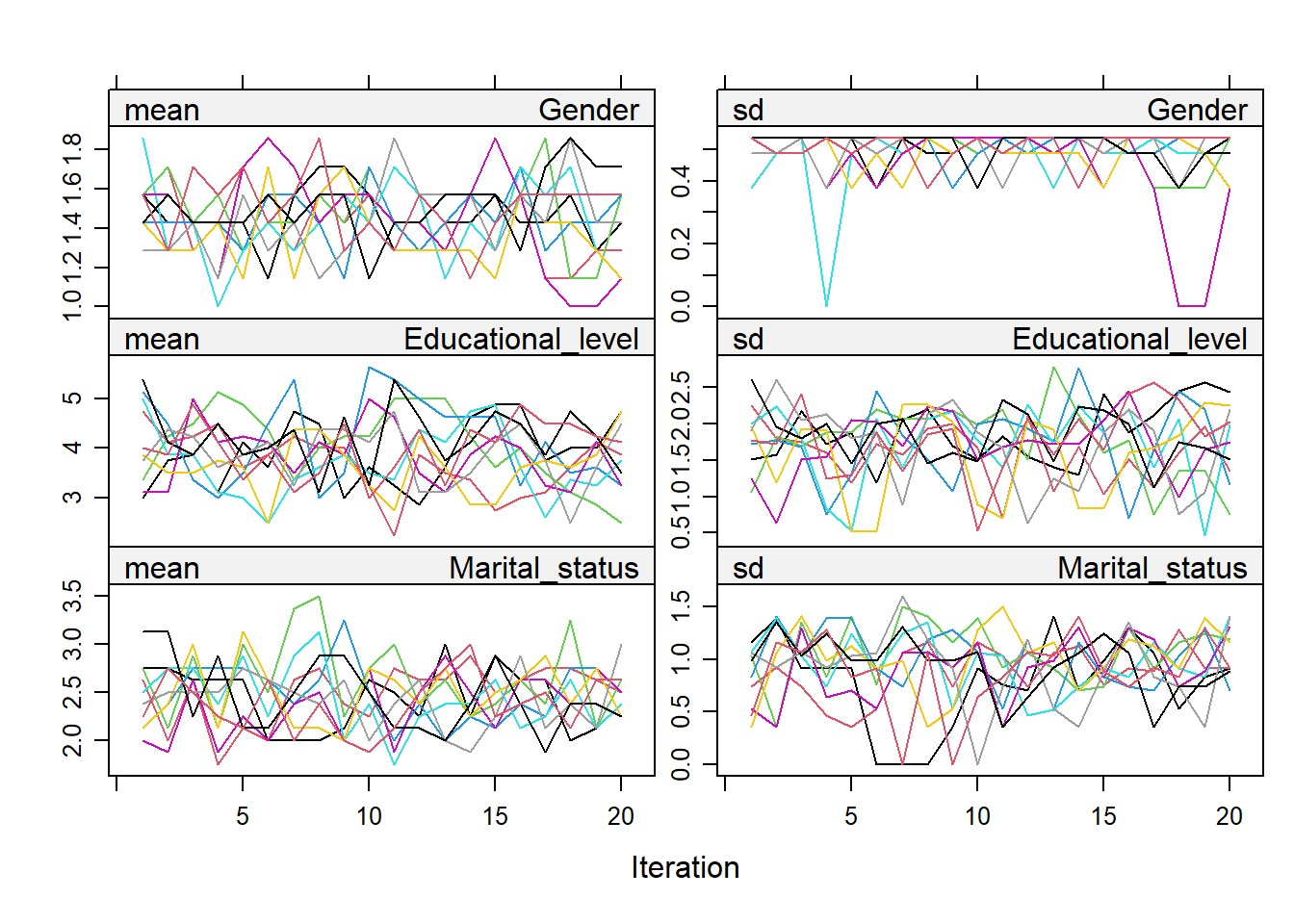


Figure 3 - Convergence plot


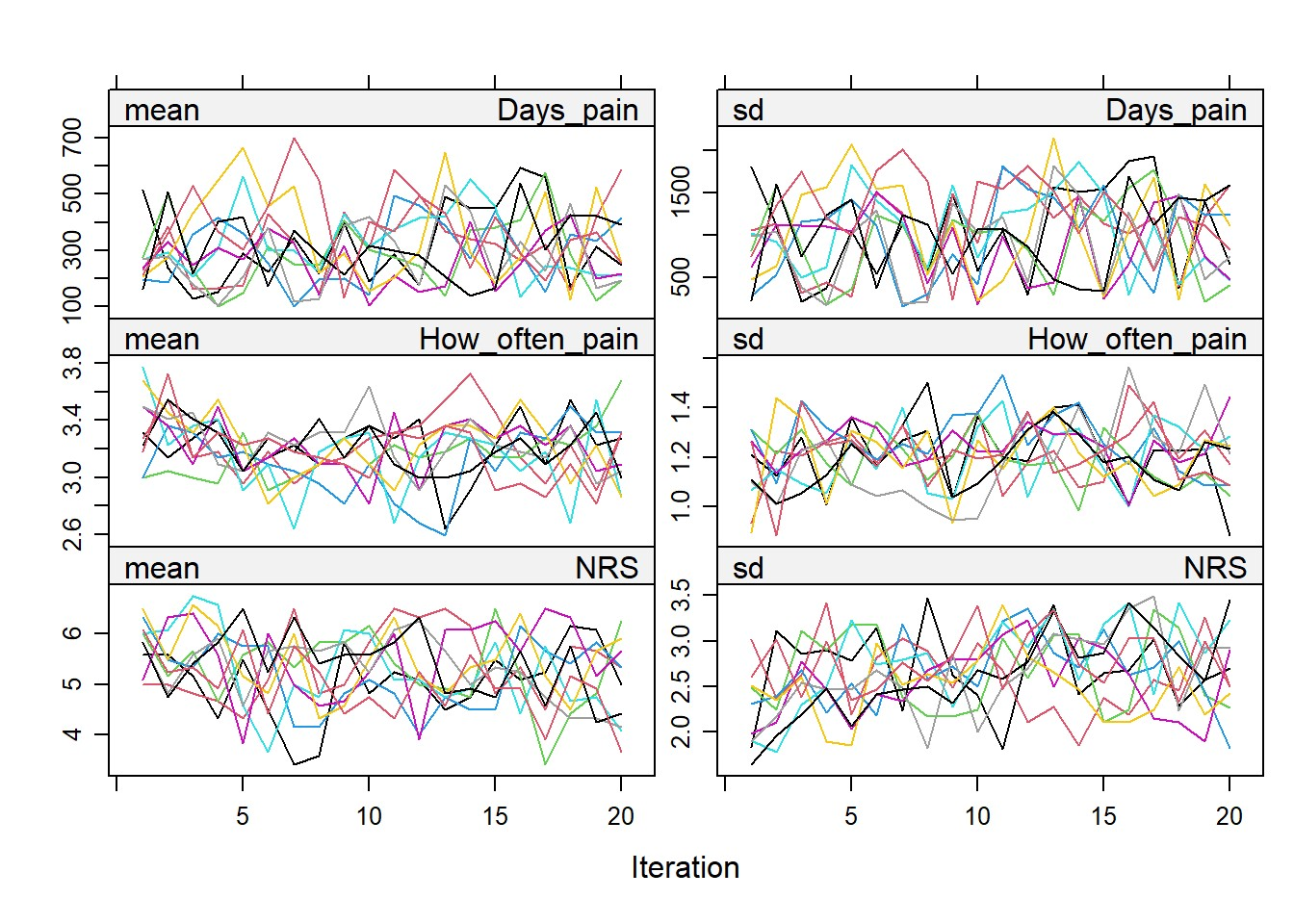


Figure 4 - Convergence plot


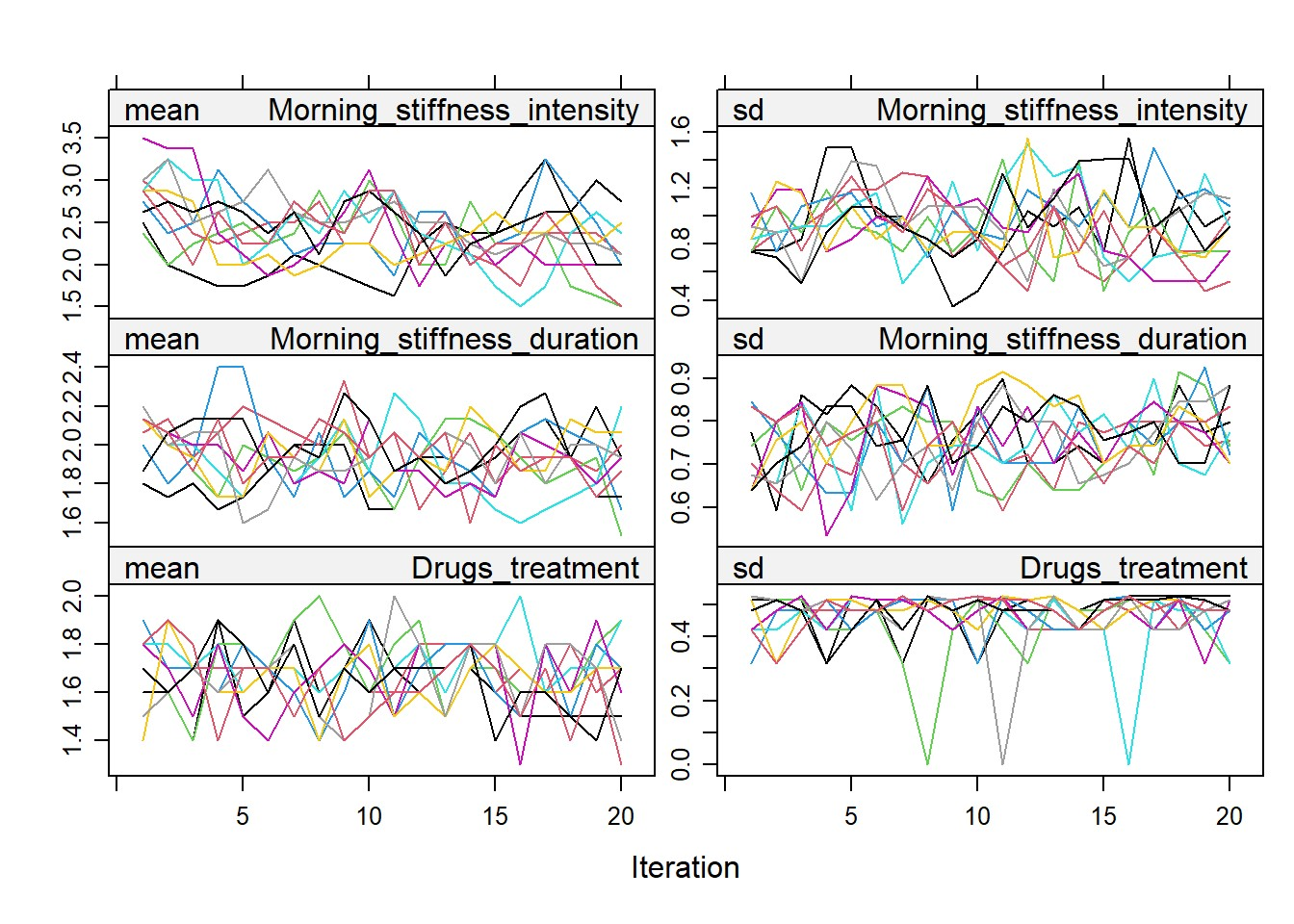


Figure 5 - Convergence plot


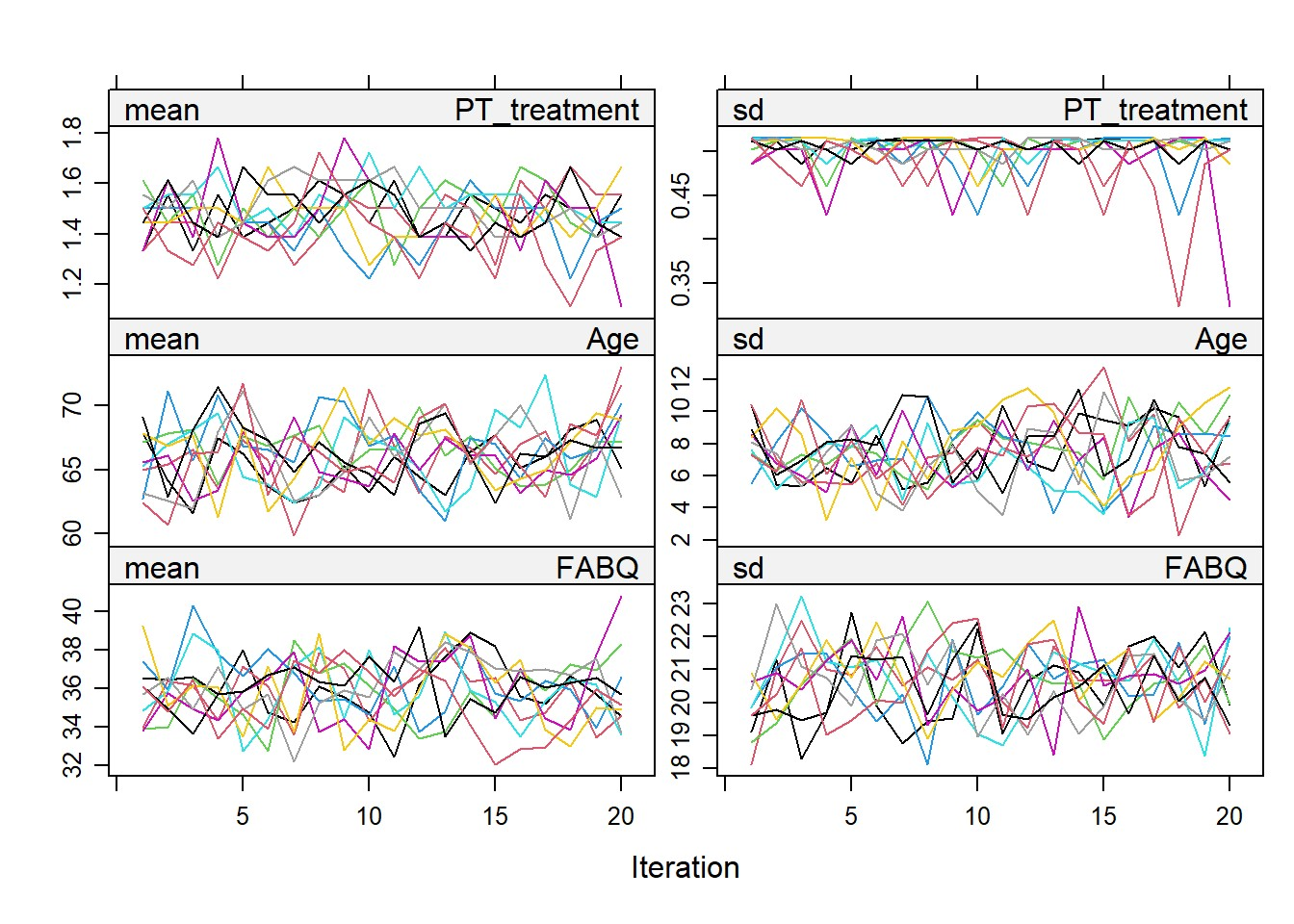


Figure 6 - Convergence plot


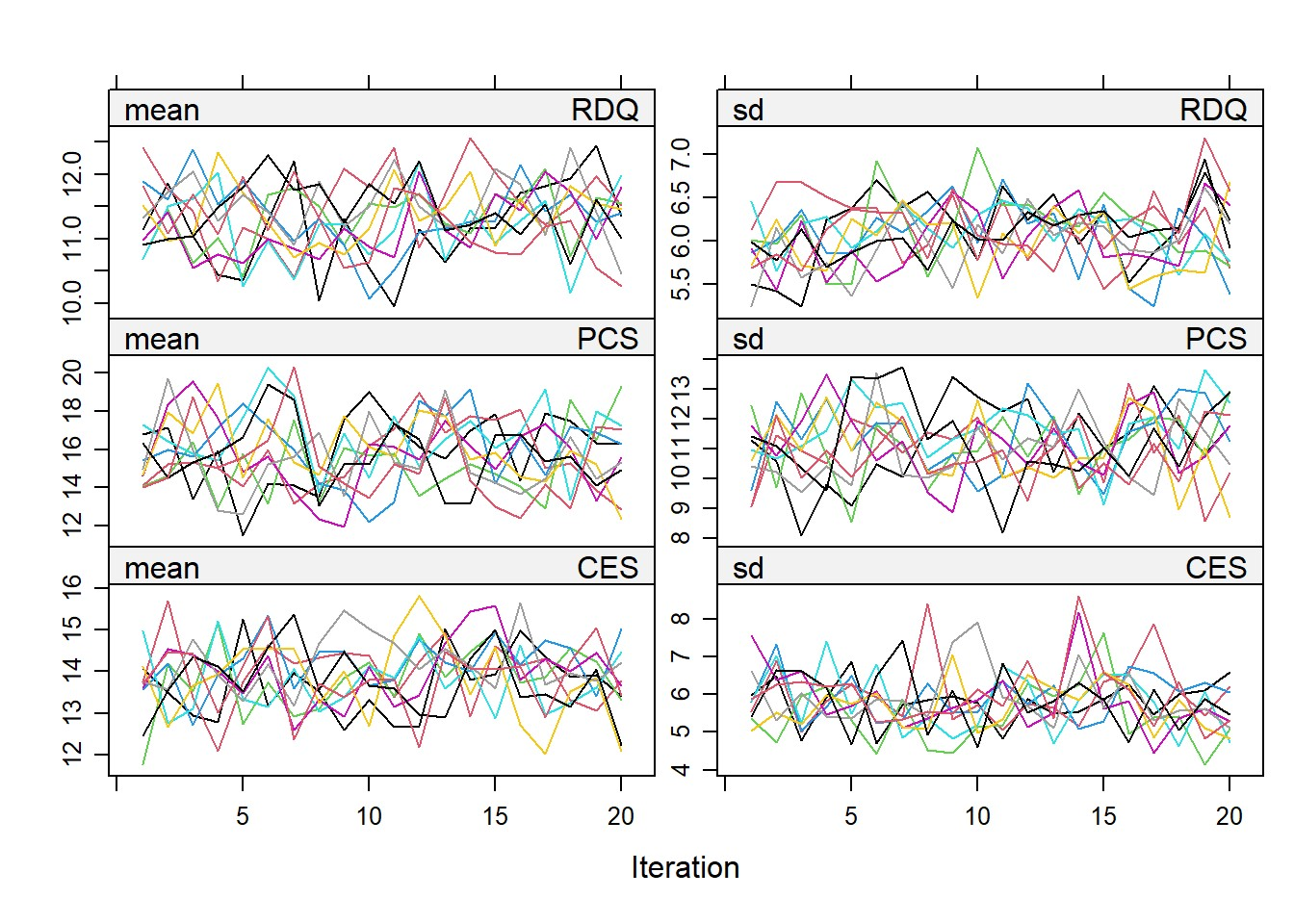


Figure 7 - Convergence plot


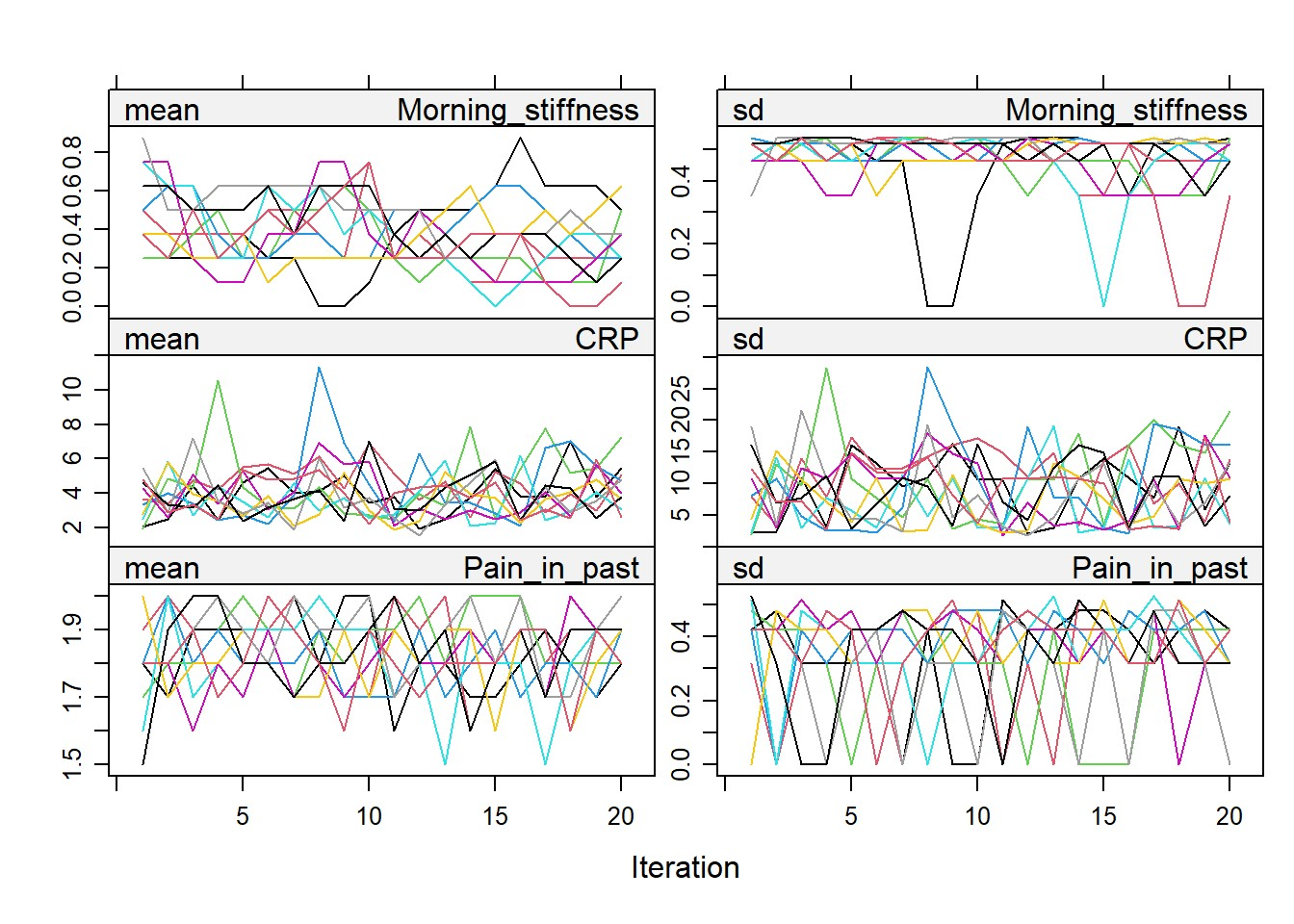


Figure 8 - Convergence plot


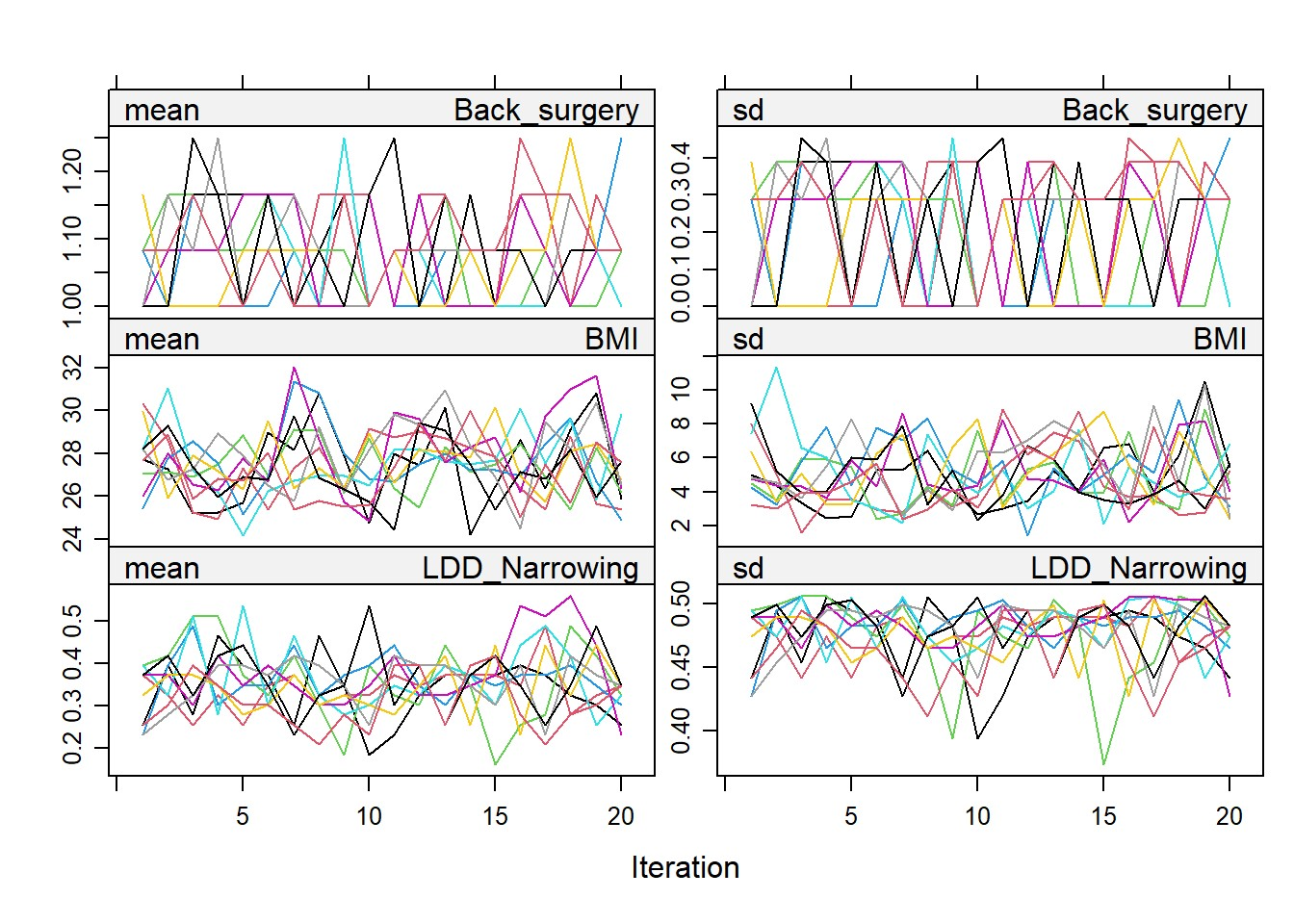


Figure 9 - Convergence plot


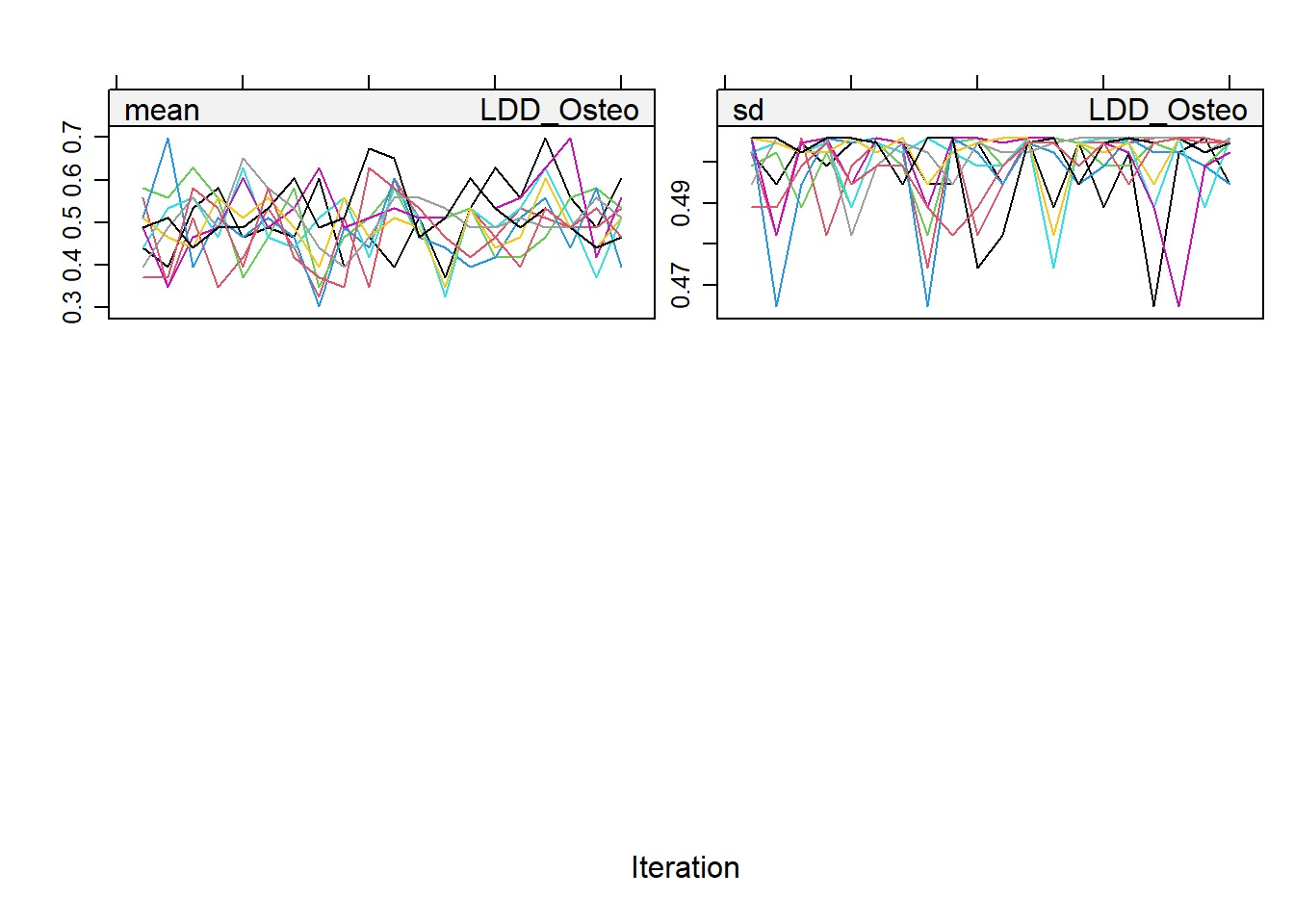


Figure 10 - Convergence plot


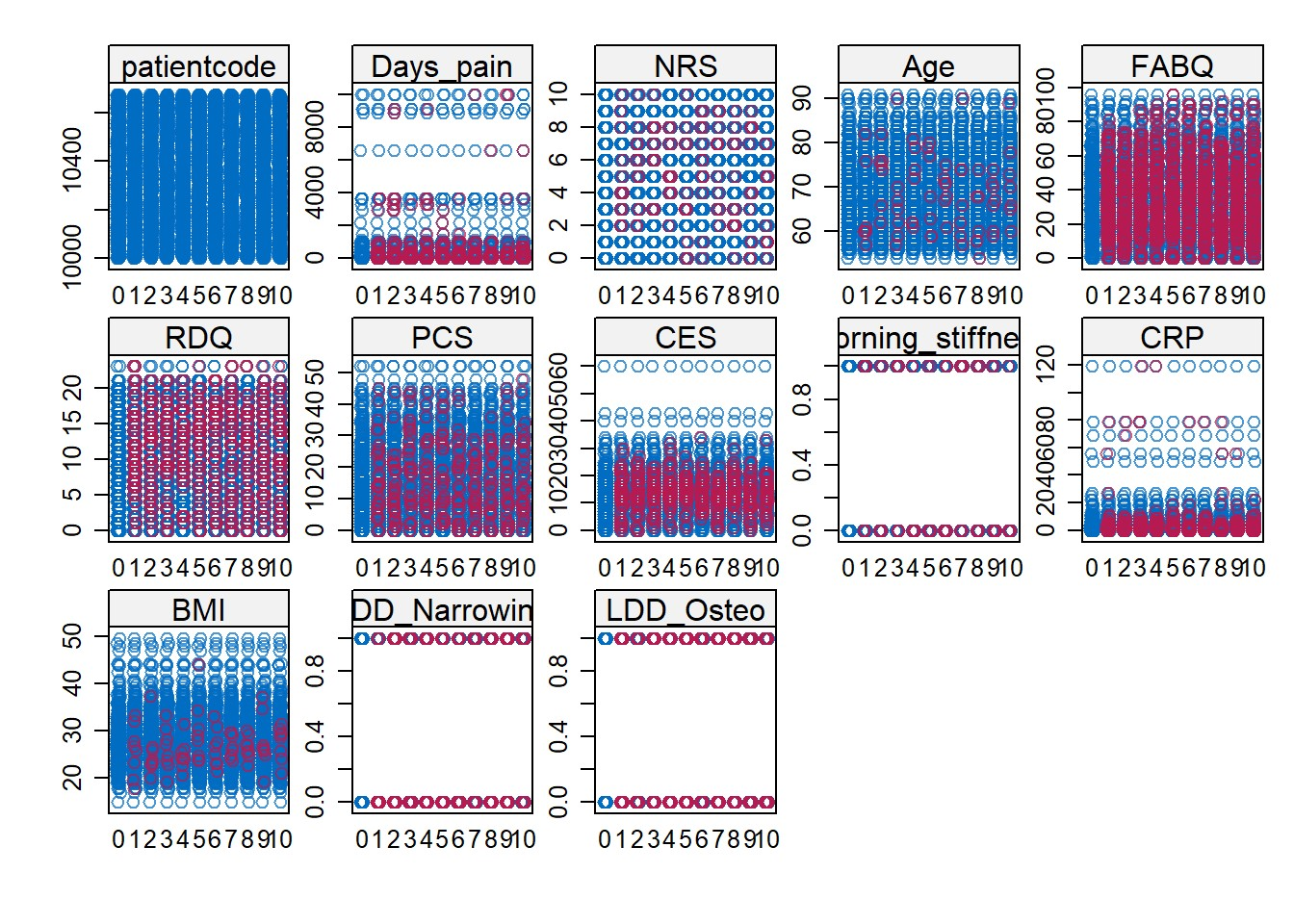


Figure 11 - Multiple imputation diagnostics

In all the models, we assessed the assumption of the absence of multicollinearity using the Variance Inflation Factor with a cut-off of 5. ^2^ In addition, we evaluated the lack of influential observations using the DFBETAS with a cut-off of 2/$\sqrt{n}$. ^2^

**REFERENCES**

1. Buuren S van. *Flexible Imputation of Missing Data, Second Edition*. 2nd ed. Chapman and Hall/CRC; 2018. doi:10.1201/9780429492259

2. Harrell , FE. *Regression Modeling Strategies: With Applications to Linear Models, Logistic and Ordinal Regression, and Survival Analysis*. Springer International Publishing; 2015. doi:10.1007/978-3-319-19425-7
