# Supplementary material for: The association of spinal morning stiffness with lumbar disc degeneration and C-reactive protein: The back complaints in older adults (BACE) study
Source: Osteoarthr Cartil Open. 2024 Oct 18;6(4):100535. doi: 10.1016/j.ocarto.2024.100535 (PMC11566339; doi:10.1016/j.ocarto.2024.100535)
Supplement: Multimedia component 2 [file mmc2.docx]

|  | **MULTILEVEL DISC SPACE NARROWING AND OSTEOPHYTES** | | |
| --- | --- | --- | --- |
|  | **Not missing** | **Missing** | **P-value** |
| **Morning stiffness severity** |  |  |  |
| None | 87 (0.95) | 5 (0.05) | 0.64 |
| Mild | 150 (0.97) | 5 (0.03) |  |
| Moderate | 214 (0.93) | 15 (0.07) |  |
| Severe | 137 (0.93) | 10 (0.07) |  |
| Extreme | 42 (0.93) | 3 (0.07) |  |
| **Morning stiffness duration** |  |  |  |
| No morning stiffness | 155 (0.95) | 8 (0.05) | 0.06 |
| Less than 30 minutes | 324 (0.96) | 14 (0.04) |  |
| More than 30 minutes | 145 (0.91) | 15 (0.09) |  |
| **CRP** |  |  |  |
|  | 3.0 (6.2) | 6.3 (19.9) | 0.01 |
| **NRS** |  |  |  |
|  | 5.1 (2.7) | 5.6 (2.6) | 0.27 |
| **Gender** |  |  |  |
| Females | 369 (0.94) | 25 (0.06) | 0.47 |
| Males | 262 (0.95) | 13 (0.05) |  |
| **Age** |  |  |  |
|  | 66.4 (7.6) | 68.2 (9.5) | 0.18 |
| **BMI** |  |  |  |
|  | 27.5 (4.7) | 28.1 (4.7) | 0.45 |

**SUPPLEMENTARY 2 – COMPARISON MISSING AND NON – MISSING OBSERVATIONS**
