# Supplementary material for: The association of spinal morning stiffness with lumbar disc degeneration and C-reactive protein: The back complaints in older adults (BACE) study
Source: Osteoarthr Cartil Open. 2024 Oct 18;6(4):100535. doi: 10.1016/j.ocarto.2024.100535 (PMC11566339; doi:10.1016/j.ocarto.2024.100535)
Supplement: Multimedia component 3 [file mmc3.docx]

|  | DISC SPACE NARROWING | | OSTEOPHYTES | | STIFFNESS SEVERITY | | STIFFNESS DURATION | |
| --- | --- | --- | --- | --- | --- | --- | --- | --- |
|  | **Multivariable association (N = 578) ^1^** | | **Multivariable association (N = 578) ^1^** | | **Multivariable association**  **(N = 615) ^2^** | | **Multivariable association**  **(N = 606) ^2^** | |
|  | **OR (95% CI)** | **P-value** | **OR (95% CI)** | **P-value** | **Coefficient (95% CI)** | **P-value** | **Coefficient (95% CI)** | **P-value** |
| **Morning stiffness intensity** |  |  |  |  |  |  |  |  |
| Reference: None | **-** | **-** |  |  |  |  |  |  |
| Mild | **2.60 (1.07 to 6.57)** | **0.04** | 1.20 (0.56 to 2.57) | 0.63 |  |  |  |  |
| Moderate | 2.46 (0.90 to 6.90) | 0.08 | 2.01 (0.86 to 4.81) | 0.11 |  |  |  |  |
| Severe | 2.20 (0.73 to 6.79) | 0.16 | 1.06 (0.41 to 2.76) | 0.90 |  |  |  |  |
| Extreme | **4.65 (1.28 to 17.30)** | **0.02** | 0.77 (0.22 to 2.57) | 0.67 |  |  |  |  |
| **Morning stiffness duration** |  |  |  |  |  |  |  |  |
| Reference: No stiffness | - | - | - | - |  |  |  |  |
| Less than 30 minutes | 0.83 (0.41 to 1.72) | 0.62 | 0.62 (0.32 to 1.19) | 0.15 |  |  |  |  |
| More than 30 minutes | 1.27 (0.55 to 2.98) | 0.58 | 1.48 (0.67 to 3.26) | 0.33 |  |  |  |  |
| **CRP** |  |  |  |  |  |  |  |  |
| Spline |  |  |  |  | 0.08 (-0.06 to 0.23) | 0.26 | 0.04 (-0.11 to 0.21) | 0.55 |
| Spline’ |  |  |  |  | -0.07 (-0.26 to 0.11) | 0.42 | -0.05 (-0.25 to 0.15) | 0.63 |

**SUPPLEMENTARY 3 – COMPLETE CASE ANALYSIS**

^1^: adjusted for gender, body mass index (splines with 3 knots), age (splines with 3 knots), CRP (splines with 3 knots), mean pain last week (splines with 3 knots), duration of back pain (splines with 3 knots), and previous back surgery

^2^: adjusted for gender, body mass index (splines with 3 knots), age (splines with 3 knots), mean pain last week (splines with 3 knots), duration of back pain (splines with 3 knots), and previous back surgery

CRP: C-reactive protein
